# The Effect of α-, β- and γ-Cyclodextrin on Wheat Dough and Bread Properties

**DOI:** 10.3390/molecules26082242

**Published:** 2021-04-13

**Authors:** Anne-Sophie Schou Jødal, Tomasz Pawel Czaja, Frans W. J. van den Berg, Birthe Møller Jespersen, Kim Lambertsen Larsen

**Affiliations:** 1 Section of Chemistry, Department of Chemistry and Bioscience, Aalborg University, DK-9220 Aalborg, Denmark; asj@bio.aau.dk; 2 Lantmännen Unibake Denmark, DK-8700 Horsens, Denmark; 3 Department of Food Science, Faculty of Science, University of Copenhagen, DK-1958 Frederiksberg, Denmark; tomasz.czaja@food.ku.dk (T.P.C.); fb@food.ku.dk (F.W.J.v.d.B.); bm@food.ku.dk (B.M.J.); 4 Department of Chemistry, University of Wrocław, 50-383 Wrocław, Poland

**Keywords:** cyclodextrins, alveograph, wheat dough, bread staling

## Abstract

Cyclodextrins (CDs) are cyclic oligosaccharides that have found widespread application in numerous fields. CDs have revealed a number of various health benefits, making them potentially useful food supplements and nutraceuticals. In this study, the impact of α-, β-, and γ-CD at different concentrations (up to 8% of the flour weight) on the wheat dough and bread properties were investigated. The impact on dough properties was assessed by alveograph analysis, and it was found that especially β-CD affected the viscoelastic properties. This behavior correlates well with a direct interaction of the CDs with the proteins of the gluten network. The impact on bread volume and bread staling was also assessed. The bread volume was in general not significantly affected by the addition of up to 4% CD, except for 4% α-CD, which slightly increased the bread volume. Larger concentrations of CDs lead to decreasing bread volumes. Bread staling was investigated by texture analysis and low field nuclear magnetic resonance spectroscopy (LF-NMR) measurements, and no effect of the addition of CDs on the staling was observed. Up to 4% CD can, therefore, be added to wheat bread with only minor effects on the dough and bread properties.

## 1. Introduction

Cyclodextrins (CDs) are cyclic non-reducing starch derivatives made by enzymes. The most commonly applied CDs are α-, β-, and γ-CD, which are cyclic oligosaccharides consisting of 6, 7, and 8 glucopyranose units, respectively. These three CDs are widely applied, as they have several beneficial attributes in, e.g., pharmaceuticals [1,2,3], foods [4,5,6,7,8], and cosmetics [9]; moreover, various health benefits have been observed when they are consumed [5,10]. Many of these effects originate from the ability of the CDs with their relatively hydrophobic cavity to form inclusion complexes with primarily lipophilic compounds or compounds with lipophilic moieties and thereby change the apparent properties of these [1,7]. When the CDs are applied in breadmaking, previous studies have found positive effects on bread quality (assessed by loaf volume) [11,12,13] and bread staling behavior [14,15]. In other food products, CDs are used as carriers and stabilizers of functional compounds, and they have, therefore, found multiple applications related to the extension of shelf life, food processing, and sensory improvement of food products [4,5,6,7,8].

Native α-, β-, and γ-CD are all considered non-toxic and safe for human consumption and have, therefore, received GRAS status (generally recognized as safe) [5,7]. The ADI (allowed daily intake) of α- and γ-CD is unspecified, while β-CD has been allocated with an ADI of 0–0.5 mg/kg body weight [16,17,18,19,20,21,22,23]. The approved use levels for bread, rolls, cakes, baking mixes, and refrigerated doughs have been set at 5, 2, and 1% (*w*/*w*) for α-, β-, and γ-CD, respectively [21,22,23].

CDs have multiple health effects, which makes them useful bioactive food supplements and nutraceuticals. There are various potentials in the application of CDs in bread, as bread is one of the most frequently consumed cereal products and a large source of available carbohydrates in the diet [24]. α- and β-CD can be considered as dietary fibers for controlling body weight and blood lipid profile, as the digestibility of α- and β-CD by the (human) amylolytic enzymes in the human gastrointestinal tract is negligible, while γ-CD is readily degraded [5,25,26,27]. α- and β-CD are instead partly fermented by gut microflora, and they have shown to be prebiotics that are able to improve the intestinal microflora [28]. Supplementation of CDs to starchy food has been shown to reduce their glycaemic index [29,30,31,32,33], which is considered favorable to health [24]. Health claims related to α-CD as dietary fiber and its ability to reduce post-prandial glycaemic responses have been permitted by The European Food Safety Authority [10].

A number of studies have investigated the effects of the addition of up to 3% pure CD of the flour weight on the wheat dough and bread performance [11,12,13,14,15]. The addition of CDs has been shown to change the mixing properties of the dough by increasing the water absorption and affecting the dough development time [11,13,15], increase the bread volume as well as texture and crumb structure with the addition of CD up to a certain concentration [11,12,13,14], and decrease the staling of the bread [14,15]. The effects of the addition of CD producing amylolytic enzymes (specifically cyclodextrin glycosyltransferases, CGTases, of various origin) on the properties of wheat bread have been investigated in various studies [34,35,36,37]. Here the CGTases were found to improve selected properties, including specific volume, texture, and staling rate of the resulting bread on par or superior to other amylolytic enzymes (e.g., commercial anti-staling enzymes). Some studies have attempted to quantify the amount of α-, β-, and γ-CDs produced by a CGTase from gluten-free baked bread [38,39] and found concentrations of up to around 59 mg CD/g crumb (sum of α-, β-, and γ-CD) [39].

As judged from the literature, immediate positive effects of supplementing industrial bread with CDs are improved bread volume and a pronounced anti-staling effect, which are key parameters for bread quality. However, the bigger potential for supplementation of wheat bread with CD may lie in their nutraceutical properties, including glycemic index reduction, as well as their prebiotic, anti-obesity, and anti-diabetic effects. Nevertheless, although previous studies have revealed potential positive effects relative to bread quality and shelf life, it is also evident that there is a limit to the amounts of a particular CD that can be supplemented to a wheat bread without compromising the key quality parameters of the products, processing suitability of the dough, and final product quality. In order to elucidate the effects of the CDs on processability and product quality of simple wheat bread, we have conducted a comparative study of the effects of the addition of α-, β-, and γ-CD in the range of 1 to 8% relative to wheat flour on both dough properties and bread quality. Dough properties were determined using alveograph and consistograph analysis, while bread properties were assessed by specific bread volume and staling measurements by texture analysis and low field nuclear magnetic resonance (LF-NMR).

## 2. Results and Discussion

### 2.1. Effect on Dough Properties

**Figure 1:**
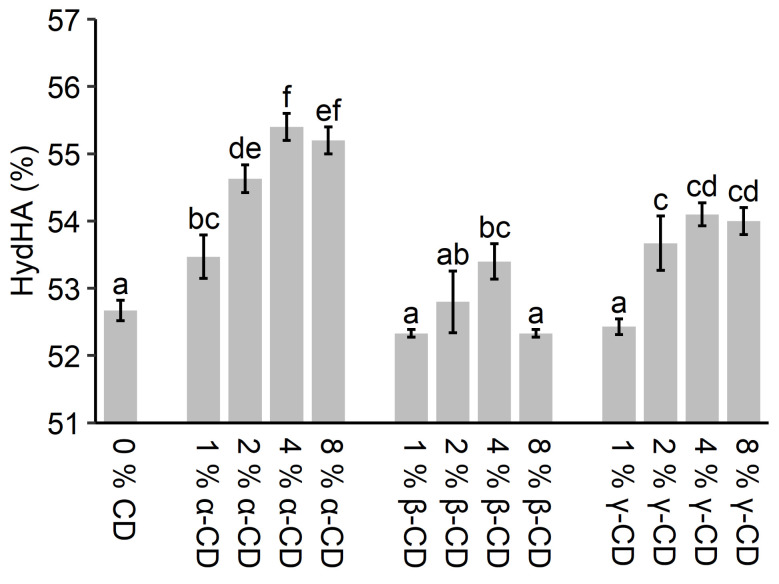
Water absorption for different types and concentrations of CD determined by the consistograph method. The water absorption is the hydration needed to obtain a dough with a maximum pressure of 2200 mbar. The error bars indicate the standard deviation. Different letters indicate the significant difference between the treatments (*p* < 0.05).

The effect of the α-, β-, and γ-CDs on flour water absorption was investigated by consistograph analysis, and the results can be seen in Figure 1. The water absorption increased when up to 4% CD was added after which water absorption was approximately constant or decreased again. The water absorption of the control dough (52.7%) increased the most by addition of 4% α-CD (55.4%), while it increased less for 4% γ-CD (54.1%) and 4% β-CD (53.4%). The decrease in water absorption at 8% CD compared to 4% CD was only significant for β-CD. In general, increased water absorption was expected with increasing CD concentration, as the CDs were added to a constant amount of flour, and the total mass (dry matter) did, therefore, increase. However, the water absorption did not increase proportionally to the amount of CD added, and it stagnated or decreased when going from 4% to 8%, dependent on the type of CD.

Similar tendencies have been found by other authors. Up to 3.4% increase in water absorption dependent on the CD concentration was observed for wheat doughs supplemented with up to 1.6% β-CD by Kim and Hill [11]. Likewise, Zhou et al. [13] observed up to 6.4% increase in water absorption with increasing CD concentration of up to 3.0% α- or γ-CD for a durum wheat flour dough. Of particular interest, Duedahl-Olesen et al. [15] found that an increase in water absorption by 7.3% and 8.0% for the addition of 3% α- and γ-CD, respectively, whereas a much lower increase in water absorption, was recorded when supplying glucose and maltooligosaccharides at the same level (wt%).

**Figure 2:**
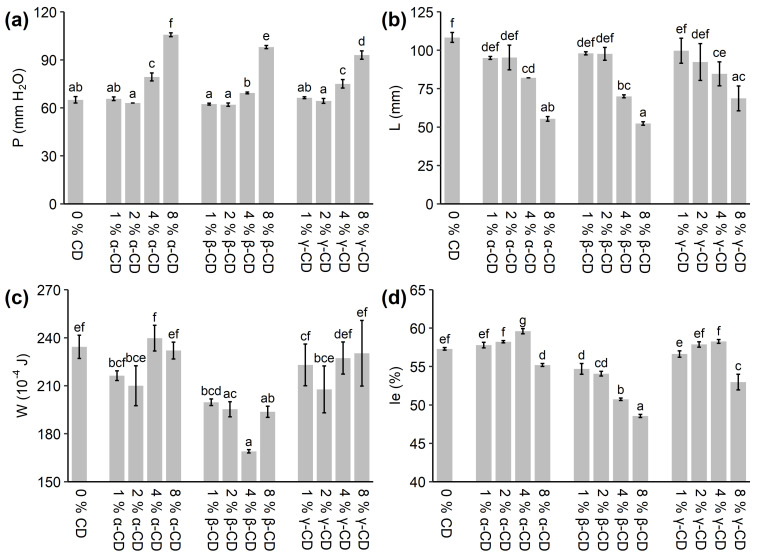
Effect of the different types and concentrations of CD on the dough extensional properties as determined by the alveograph method. The results for the alveograph parameters *P* (**a**), *L* (**b**), *W* (**c**), and *Ie* (**d**) are shown. The error bars indicate the standard deviation. Different letters indicate the significant difference between the treatments (*p* < 0.05).

The effects of the different concentrations of α-, β-, and γ-CD on the biaxial extensional properties of the dough were tested by alveograph analysis. The results can be seen in Figure 2. The *P* value, which represents the tenacity of the dough, was approximately constant up to 2% CD (no significant difference from the control), after which it increased with increasing CD concentration for all three types of CD. A concentration of 8% α-, β-, and γ-CD caused an increase in *P* of 63%, 51%, and 43%, respectively. The biaxial extensibility of the dough measured by the *L* values decreased with increasing concentration of the three types of CDs. 8% addition of α-, β-, and γ-CD caused a decrease in *L* of 49%, 52%, and 37%, respectively. The deformation energy measured by the parameter *W* seemed to decrease for low concentrations of CDs, after which it increased again for higher CD concentrations. However, the decrease in *W* was only significant for β-CD, while the *W* values for α- and γ-CD supplemented doughs were not significantly different from the control for any concentration. The *W* value for all concentrations of β-CD was significantly below the value of the control. The *Ie* value, which is called the elasticity index, changed differently dependent on whether α- and γ-CD or β-CD were applied. The *Ie* value seemed to increase for a concentration of up to 4% α- and γ-CD. However, only the *Ie* value for 4% α-CD was significantly different from the control. At 8% α- and γ-CD, the *Ie* value decreased significantly. The *Ie* value decreased with increasing concentration of β-CD. The alveograph results reveal that addition of CDs entails a stiffer and less extensible dough, as *P* increases, while *L* decreases. Addition of α- and γ-CD did not change the strength of the dough significantly, as indicated by *W*, while addition of β-CD resulted in a weaker dough. This was further substantiated by the *Ie* values obtained for the β-CD series of doughs, as according to Kitissou [40], *Ie* is related to the gluten network quality of the dough. However, the addition of 8% α- and γ-CD also resulted in a significant decrease of the *Ie* value.

While a few studies have investigated the effect of CDs on the mixing properties of the dough, the effect on the extensional properties of the dough has only been studied to a limited extent. Zhou et al. [12] investigated the effect of β-CD on the dough using the extensograph. They found that 0.5–1.5% β-CD increased the maximum resistance to deformation compared to the control, while the maximum resistance to deformation decreased for 2.0–3.0% β-CD. The extensibility increased slightly up to 1.0% β-CD, after which it decreased slightly up to 3.0% β-CD. The results from the alveograph method and the extensograph method cannot be directly compared due to differences in the sample and analysis conditions. However, the study by Zhou et al. [12] supports that at least elevated amounts of β-CD resulted in a weaker dough, probably through a weakening of the gluten network.

The addition of CD to wheat dough has multiple effects, as the CDs can affect both water distribution and the other flour constituents. The water in wheat dough interacts with the different constituents of dough, but the water availability is in general limited [41]. CDs contain multiple hydroxyl groups, which are able to form hydrogen bonds with the water. The addition of CDs might, therefore, limit the availability of water and thereby affect the gluten network development, which would be observed as changes in the mixing and extensional properties of the dough, including the HydHA value and the alveograph parameters. However, our results and other studies indicate that the effects of CDs are also caused by their direct influence on other components of the dough matrix and not just a shift in the distribution of water. Duedahl-Olesen et al. [15] found that α- and γ-CD resulted in higher water absorption during mixing compared to an equal amount of glucose or non-cyclic maltooligosaccharides. If the higher water absorption should only be attributed to the water-binding capacity of the hydrophilic CDs, similar effects should be expected using their non-linear counterparts, as the water binding capacity is considered to be comparable in the dough matrix with its limited water availability. Furthermore, in the alveograph analysis, doughs with similar consistencies according to the HydHA values were analyzed, which was considered to reduce the effect of the varying water absorption on the results. This suggests that the large changes that were observed in the resultant parameters cannot solely be explained by differences in varying water absorption.

One characteristic that distinguishes CDs from smaller carbohydrates and, to some extent starches, is their general ability to form inclusion complexes by exchanging water in the cavity with a hydrophobic molecule or part of a molecule. In this process, complex formation is mainly driven by the release of “enthalpy rich” cavity-bound water and hydrophobic interaction (removal of ordered low entropy, high enthalpy water around the hydrophobic guest) [42]. Both driving forces would be expected to be favorable in an environment with low water activity. The CDs are (relatively rigid) cyclic oligosaccharides, and they are, therefore, capable of forming rather stable inclusion complexes with a range of primarily lipophilic molecules [1,2,5]. The CDs might interact with lipophilic molecules (e.g., lipids) and lipophilic parts of molecules, e.g., lipophilic parts of gluten proteins, but the strength and selectivity will be dependent on the cavity size of the specific CD. In essence, α-CD is most suitable for complex formation with linear aliphatic molecules (such as lipids), β-CD is suitable for complex formation with aromatic molecules, and γ-CD is suitable for larger aromatic molecules [1,2,5]. Although this leads to a considerable degree of selectivity, the complex-forming ability of the CDs is somewhat general, as typically all three CDs will be able to form a complex with a given (preferably lipophilic) molecule, but with different association constants.

The starch in the dough might also be affected by the addition of CDs. It has previously been suggested that β-CD might disrupt the amylose-lipid complex formation as well as it might form amylose-β-CD and amylose-lipid-β-CD complexes [14,43,44,45]. This might change the crystallinity of the starches and thereby cause an indirect change in the distribution of water, which has been suggested to affect the mixing properties [15]. However, the disruption of amylose-lipid complex formation and formation of complexes with CDs have primarily been observed for starch, which was at least partly gelatinized. In the dough, most of the starch is organized in starch granules, and the accessibility of the starch is, therefore, limited [46]. The effects of interactions between CDs and starch are, therefore, assumed to be smaller for the dough compared to the bread where the starches have been subjected to extensive gelatinization.

The CDs might also interact with the gluten proteins in the dough due to their ability to form weak inclusion complexes with proteins, which might affect the development and the properties of the gluten network. This was identified by Zhou et al. [12], who found that the addition of β-CD to wheat dough changed the secondary structure of the gluten proteins by increasing the proportion of α-helixes and decreasing the proportion of β-sheets. α-, β-, and γ-CD have been shown to be able to influence the behavior of proteins [47,48,49,50], but β-CD causes by far the largest effects, which have been explained by a relatively large affinity towards solvent exposed aromatic amino acids [47,51]. The interaction between β-CD and aromatic amino acids reduces the formation of protein-protein interactions by hydrophobic interaction in aqueous solutions [47,48]. All CDs had a significant influence on the viscoelastic properties of the dough, but especially β-CD was revealed to have large effect on the value of the alveograph parameters *W* and *Ie*, which are among other things dependent on the gluten network quality. These effects might be caused by interaction between the gluten proteins and β-CD, leading to changes in the strength of potential protein-protein interactions by non-covalent interactions, including hydrophobic interactions. The results indicate that the addition of a large amount of β-CD leads to a lower gluten network quality as assessed from the rheological properties. However, Zhou et al. [12] suggested that the addition of up to 1.5% β-CD positively affected the gluten network, as the maximum dough tensile resistance in extension increased. In addition to the effects of β-CD, the addition of CDs dilutes the protein content in the dough, which might also decrease the strength of the gluten network. This might be a contributing cause to why the addition of 8% of any of the CDs results in a lower than expected increase in the water absorption as well as the low *Ie* values in the alveograph analysis.

### 2.2. Effect on Bread Properties

**Figure 3:**
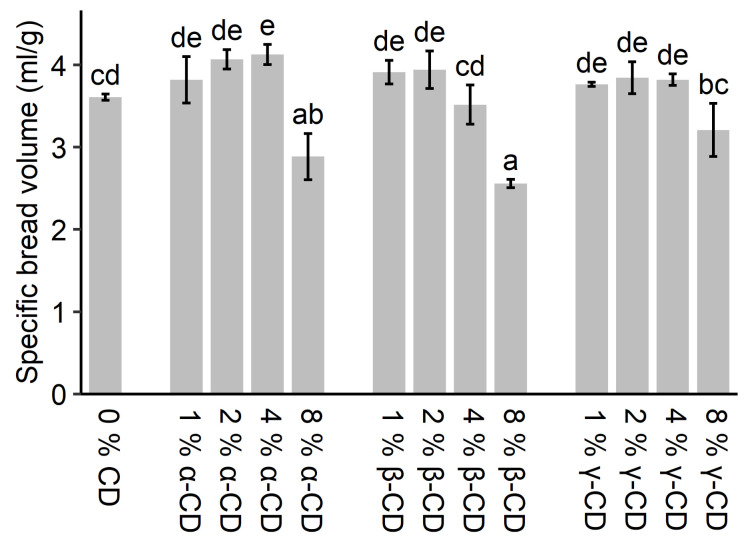
Specific bread volume for wheat bread with the addition of different types and concentrations of CD (Breadmaking I). The error bars indicate the standard deviation. Different letters indicate the significant difference between the treatments (*p* < 0.05).

Baking experiments with up to 8% addition of α-, β-, and γ-CD were made on a domestic bread maker (Breadmaking I) to investigate the effect on the bread volume. The results can be seen in Figure 3. Although the addition of CDs affected the water absorption, we decided to apply constant water addition in the bread doughs to minimize the number of variables. For the different types of CD, the largest specific bread volumes were observed for 4% α-CD, 2% β-CD, and 2% γ-CD, which resulted in an increase of 14%, 9%, and 7% in specific bread volume, respectively. However, only the bread with 4% α-CD were significantly larger than the control sample. When higher concentrations of the three types of CDs were added, the specific bread volume decreased, especially when β-CD was applied. All bread with the exception of the 8% α-CD, and 4% and 8% β-CD supplemented version, displayed acceptable crust and crumb structure as perceived by visual and manual inspection (see Supplementary Materials). In contrast, for the exceptions, it was observed that the crumb of the bread had partially collapsed, had an irregular crumb, and was very dense at the bottom. An irregular and uneven crust was observed at the top of the bread as if air had escaped. This indicates that the gluten network had been adversely effected by the addition of CD, preventing the development of a suitable gluten network with sufficient stability from supporting the dough foam.

Similar bread was produced using a kitchen mixer (Breadmaking II). 8% addition of CD was omitted, as this concentration resulted in a significant decrease in specific bread volume. During the preparation of the dough pieces for the analysis, it was noted that the dough stickiness increased with increasing CD concentration. In contrast to the bread produced in the domestic bread maker (Breadmaking I), no significant difference in specific bread volume between the bread with and without CD could be observed. The specific bread volumes in this trial ranged from 3.6–3.9 mL/g.

These results only partly confirm the results presented in other studies, where, in general, significant increases in bread volume could be observed in the range of 1–3% added CD. Kim and Hill [11] showed that an increase in bread loaf volume of 12% could be obtained in the range of 0.8 to 1.4% β-CD added for wheat bread. Mutsaers and Eijk [34] reported a 14–20% increase in loaf volume for two types of wheat bread supplemented with 1.5–2% β-CD. The addition of β-CD was found to be on par with the addition of shortening (3%), CGTase, and amylase in an American straight dough process judged from bread loaf volume [34]. Zhou et al. [12] found a slight increase in specific loaf volume until 1.5% β-CD after which the specific volume decreased below the specific volume of the control without β-CD. Zhou et al. [13] found a maximum increase in a specific volume at 2% α-CD and 3% γ-CD in a study in which the range of added CD was 0.5 to 3%. Both Zhou et al. [12] and Zhou et al. [13] observed a change in bread crumb pore distribution towards smaller and more uniform pores for the bread supplemented with either 2% α-CD, 1.5% β-CD, or 3% γ-CD. Furthermore, multiple studies have used CGTases in the production of wheat bread and found bread volume increments, which is assigned to the production of CDs [34,35,37]. Although our results, at least for the Breadmaking II data set, did not fully corroborate the data obtained on a domestic bread maker (Breadmaking I), we can partly confirm the tendency that the addition of small amounts (1–2%) CD may lead to an increase in bread volume. On the other hand, our results clearly demonstrate that the addition of larger amounts of CD, e.g., >4%, leads to a loss of bread volume compared to the control. However, the changes in bread volume are, as shown, somewhat dependent on production conditions and procedure. Although significant increases in bread volume based on the addition of CDs could not be unequivocally verified, our results underline that acceptable bread with respect to bread volume and quality may be achieved for additions of all three native CDs up to at least 4%.

The results obtained for the effects of CDs on bread volume, in essence, corroborates the tendencies obtained from the alveograph analysis of the doughs, including minor increases in parameters correlated to bread quality (volume; e.g., *Ie*) at low CD concentrations, followed by large decreases at high CD concentration. This is to some extent expected since both alveograph analysis and the foam producing step in breadmaking involves bubble inflation causing biaxial extension of the dough matrix [52]. This substantiates that the tendencies found for the effects of CD on bread volume are caused by the interaction of the CD with the proteins in the gluten network, facilitating minor improvements of the network quality (as judged by bread volume) at low concentration and larger adverse effects at high CD concentration.

To evaluate the effects of the CDs on the staling of the bread, the bread crumb from bread stored at room temperature were analyzed by texture analysis and LF-NMR to detect changes in the firmness and in the water distribution, respectively.

**Figure 4:**
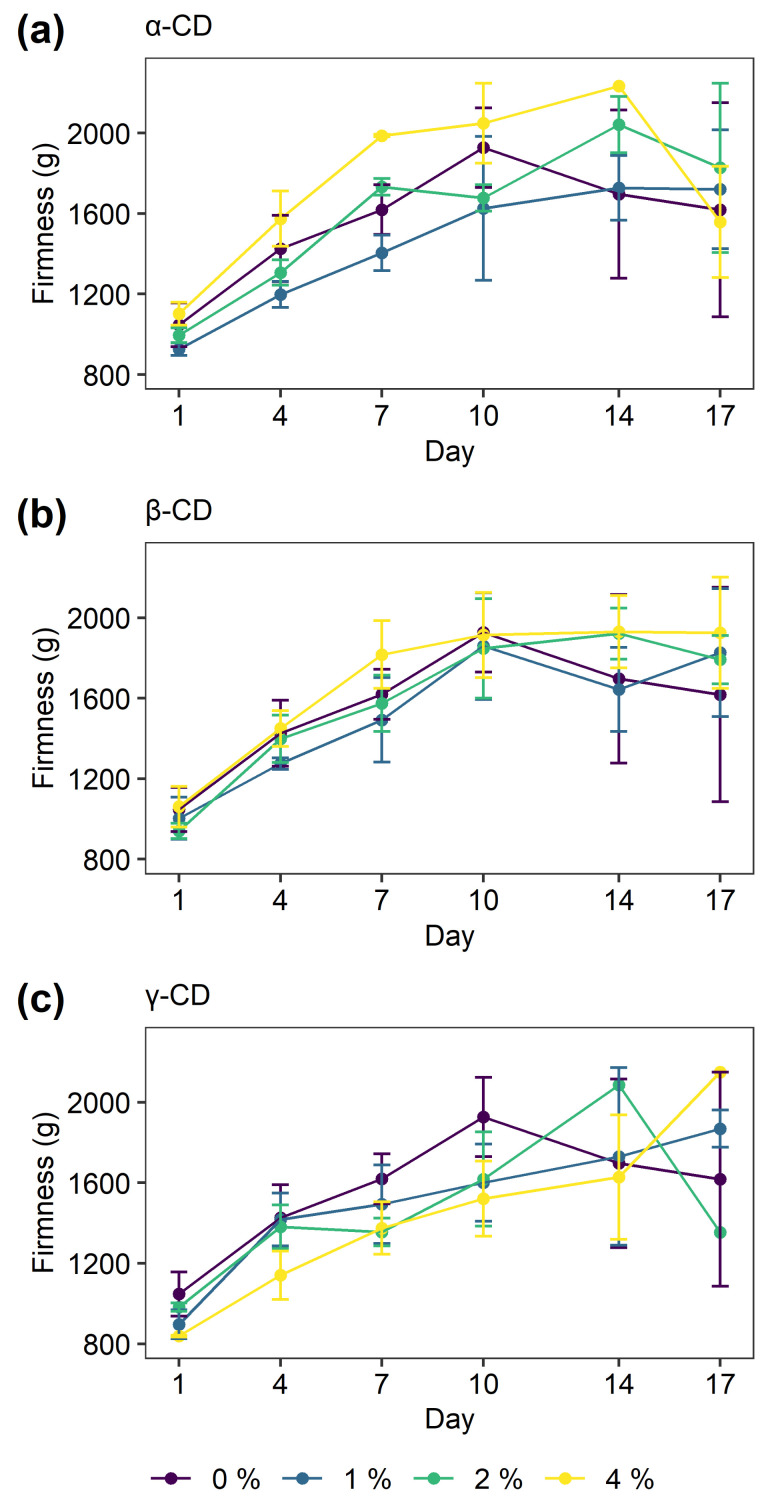
Firmness of bread without and with the addition of α-CD (**a**), β-CD (**b**), and γ-CD (**c**) for different storage time. The error bars indicate the standard deviation.

The result of the texture analysis can be seen in Figure 4. The firmness of the bread crumb gradually increased with longer storage time for all the bread. Increasing firmness of bread crumb is often used as a measure of bread staling [53]. No significant difference in the firmness measurements between the bread with and without CD during the storage could be found, indicating that the three types of CD did not retard the staling of the bread as judged by firmness.

**Figure 5:**
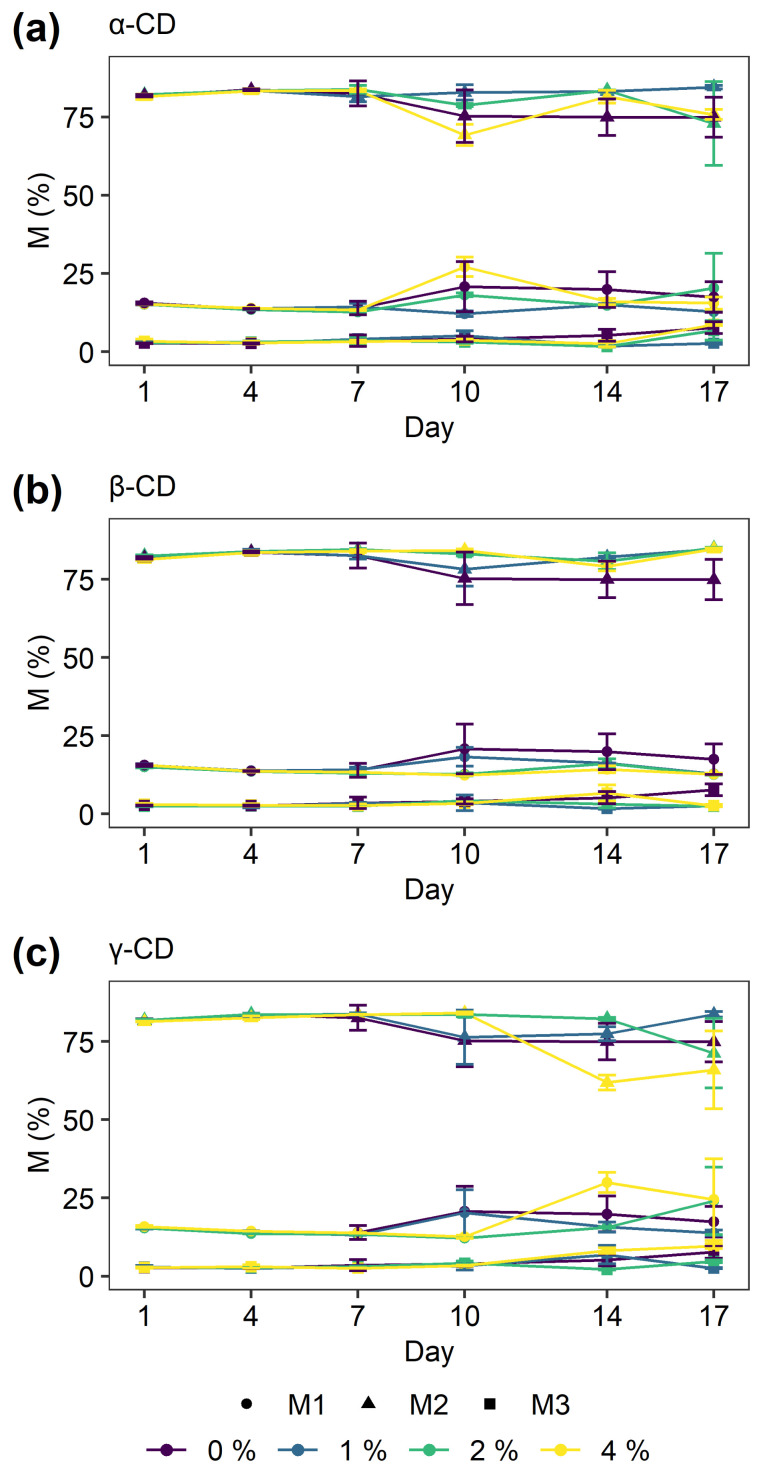
The relative abundances (M_n_) of the three different proton populations in the breads with different types and concentrations of α-CD (**a**), β-CD (**b**) and γ-CD (**c**) for different storage time. The error bars indicate the standard deviation.

To further elucidate a potential effect on staling, LF-NMR analysis on breadcrumb was conducted. The use of LF-NMR in food science is well established [54]. The LF-NMR data were analyzed to label discrete exponential decays, representing distinct water populations. Three populations of protons were identified in all bread samples with relaxation time T_21_ varying between 0.5–2.1 ms, T_22_ 2.8–7.7 ms, and T_23_ 17.4–36.6 ms (see Supplementary Materials). The ranges of T_2n_ values are similar to those presented in the literature [55,56]. The relaxation time T_21_ represents the least-mobile proton population and, therefore, the most tightly bound, and vice versa, T_23_ represents the most mobile proton population. No apparent systematic development of the T_2n_ values during storage was found. The corresponding M_n_-values, which are the abundances of the three proton populations, presented in Figure 5 indicate the relative concentration of the different proton populations. The figures show a stable distribution up until 7 days of storage for all treatments with a reproducible signal. After 7 days, the samples display considerable variation among the triplicate measurements within each treatment, suggesting variations within the bread crumb. A weak tendency of proton exchange between the two faster relaxation times, T_21_ and T_22_, is also observed, in which it should be noted that M_n_ is a relative indicator. Selective loss of water during the staling process will thus give the same impression.

No systematic change in the distribution of water populations could be observed between the control and the CD supplemented bread. This corroborates the firmness studies carried out on the same series of bread (Figure 4). However, the literature suggests that an anti-staling effects may be achieved by the addition of CDs to wheat bread. β- and γ-CD have been found to have a small (but significant) retarding effect on the staling rate of wheat bread stored at room temperature, while no significant decrease in staling has been observed for the addition of α-CD [14,15]. Tian et al. [14] suggested that retarding effect of β-CD on staling was caused by the formation of an amylose-lipid-β-CD complex, which retard the transformation of the crystalline starch types in the crumb. Furthermore, the retarding effect of β-CD on the retrogradation of various starches has been presented in several studies [43,44,57,58,59]. The addition of CGTases has also in multiple studies been shown to inhibit the staling in bread, but it is also presumed to be linked to the amylolytic activity of the enzymes and not solely the effects of the CD produced [35,36,37,38].

Despite that several studies have found an anti-staling effect by the addition of CDs in bread, no such effect could be identified in this study. This discrepancy may originate in differences in ingredients, water addition, processing methods, and method for assessing a potential anti-staling effect, as several of the abovementioned studies apply DSC measurements together with texture analysis. It may also underline that an anti-staling effect of CDs, if any, may be small and lower than the random variation of the experiments.

## 3. Materials and Methods

### 3.1. Materials

Two batches of commercial wheat flour (Lantmännen Cerealia) were obtained from Lantmännen Unibake, Hatting, Denmark. The first batch, which was used for consistography, alveography, and bread baking for volume measurement, had a moisture content of 14.2% and a protein content of 12.6% (dry matter basis). The second batch, which was used for bread baking for staling measurements, had a moisture content of 14.1% and a protein content of 14.5% (dry matter basis). The moisture content of the flour was determined according to AACC method 44–15.02 [60], and the protein content was determined by the Kjeldahl method as described in AACC method 46–11.02 [61]. α-cyclodextrin (food grade), β-cyclodextrin (pharma grade), and γ-cyclodextrin (food grade) were provided by Wacker Chemie. Sodium chloride was from VWR, and dry yeast (Lesaffre) was obtained from the local supermarket.

### 3.2. Consistographic and Alveographic Analysis

The flour was tested with and without 1, 2, 4, or 8% (of flour weight) of α-, β-, and γ-CD. The consistograph and alveograph measurements was made on AlveoLab (Chopin Technologies, Villeneuve-La-Garenne, France). All measurements were made in triplicate. The different concentrations and types of CDs were tested using the constant hydration consistograph test AACC method 54–50.01 [62]. In short, CDs were added on top of the required amount of flour, and a dough with a fixed water content was made. During mixing, the consistency of the dough was measured by monitoring the pressure on one side of the mixer. The maximum dough consistency was used to find the water absorption value HydHA, which was the hydration equivalent to a maximum pressure of 2200 mbar on the basis of 15% H_2_O (flour basis).

The flour with different CD type and concentrations was analyzed by alveography as described in the AACC method 54–30.02 [63], except the amount of flour and the water addition were based on the HydHA value determined in the consistograph analyses [64]. The parameters *P* (tenacity, related to the maximum height of the curve), *L* (biaxial extensibility, length of the curve at bubble rupture), *W* (deformation energy, related to the area under the curve), and *Ie* (elasticity index, ratio between the height of the curve at 40 mm and the maximum height) was found from the bubble inflation air pressure curves.

### 3.3. Breadmaking I

Bread with and without 1, 2, 4, or 8% (based on flour weight) of α-, β-, and γ-CD were made in triplicates using the recipe in Table 1. The bread was made on a domestic breadmaker (model 48319, Morphy Richards, Swinton, United Kingdom) using the settings program 5 (French bread), 450 g loaf, and 5 in crust darkness. In this program, the dough was kneaded for 13 min, rested for 40 min, kneaded again for 17 min, proofed for 30 min, kneaded shortly, proofed for a further 50 min, and baked for 60 min. The bread volume was measured using the mustard seed displacement method. The volume was calculated by subtracting the volume of seeds held by a container with a baked product from that of the volume of seeds without the baked product. The volume of the mustard seeds was determined by their weight using a density of 0.759 g/mL. All weights were determined on an analytical scale (Kern PFB 1200-2A, Balingen, Germany). The specific bread volume was found by dividing the bread volume with the weight of the bread.

### 3.4. Breadmaking II

Bread for assessment of staling rate was made using the recipe in Table 1, except doughs with 8% CD were omitted. Doughs were made for each type and concentration of CD and mixed for 5 min with a kitchen mixer (Kenwood Chef XL, Havant, UK) at mixing speed 3 with a final dough temperature of 26 °C. The dough was divided into 300 g pieces, rounded, and molded by hand, proofed at ambient temperature (26 °C) under a linen cover for 90 min and baked at 200 °C for 12 min in an oven (Rational SCC WE 101, Heerbrugg, Switzerland). The loaves were cooled at room temperature for 1 h, after which the breads were weighed, and bread volume was measured using the mustard seed displacement method allowing the specific bread volume to be calculated. Immediately hereafter, the breads were brushed with sodium benzoate solution, sealed in plastic bags, and stored at 19 °C for aging studies.

### 3.5. Crumb Firmness Measurements

A texture analyzer TA.XTplus (Stable Micro Systems, Surrey, UK) was used to measure force-time curves according to the AACC standard 74–09.01 [65] with modifications. At day 1, 4, 7, 10, 14, and 17, bread slices (2.5 cm thick) were compressed to a deformation level of 40% of the original sample height by a 25 mm cylindrical probe (P25) at a test speed of 1.7 mm/s. The peak force of compression was reported as firmness (g). Measurements in triplicates were used for the evaluation of the bread staling.

### 3.6. Low Field Nuclear Magnetic Resonance Spectroscopy Measurements

To identify possible differences in the water distribution (or rather proton populations) in the bread supplemented with different concentrations and types of CDs, bread samples from day 1,4,7,10,14, and 17 were analyzed by ^1^H low field nuclear magnetic resonance (LF-NMR) spectroscopy (MQR Spectro-P spectrometer, Oxford Instruments, Oxfordshire, UK, operating at 20 MHz). Approximately 1.5 g of bread crumb was sampled from a slice of bread using a cork borer and placed into a glass tube for NMR measurements. Data were collected using a Carr–Purcell–Meiboon–Gill (CPMG) sequence at 25 °C with the parameters: Recycle delay of 5 s, τ-delay of 100 µs, and 16 scans averaged. Data from 6000 echoes were acquired with a receiver gain of 5.0. All measurements were prepared in triplicates on distinct samples. Transverse relaxation times (T_2n_) of different relaxation components were obtained using an in-house MATLAB (version R2019a, The Math-Works) script designed for fitting the relaxation curves to a series of exponential decays according to Equation (1).

(1) $$I(t)=\sum_{n=1}^{N}M_{n}\cdot e^{-t/T_{2n}}$$

In which *I*(*t*) is the echo intensity as a function of relaxation time, *N* is the number of relaxation components, the transverse relaxation time for site *n* is *T_2n_*_,_ and the corresponding abundance is *M_n_. N* is determined by visual inspection of the residuals after model fitting. Each LF-NMR recording was fitted individually. All *M_n_*-values were presented as percentage of total intensity to eliminate sample size differences.

### 3.7. Statistical Analysis

Statistical analysis of the results was carried out in R (version 3.6.1., R Core Team) using analysis of variance (ANOVA) with Tukey’s multiple comparison procedure with a significance level of 5%.

## 4. Conclusions

α-, β-, and γ-CD affects the mixing and extensional properties of wheat dough dependent on concentration and CD type. β-CD displayed the largest effects, which may be caused by its potentially stronger (compared to α- and γ-CD) direct interaction with the proteins in the gluten network. The addition of up to 4% CD did not significantly affect the bread volume, in general, expect 4% α-CD, which resulted in a minor, significant increase in bread volume in one of the breadmaking trials. No significant effect of the CDs on staling of the bread could be detected. The results suggest that up to 4% CD can be added to bread with only minor effects on dough properties and without a significant decrease in the bread quality. This opens up for the use of CD supplemented wheat bread for nutraceutical purposes.

## Figures and Tables

**Table 1:** Recipe for bread used in Breadmaking I and Breadmaking II.

Ingredient	Ratio in Grams
Flour	100
Tap water	60
Sodium chloride	1
Dry yeast	0.8
α-, β-, or γ-CD	0, 1, 2, 4, or 8

## Data Availability

Data available on request to the corresponding author.

## Supplementary Materials

Pictures of CD-supplemented breads and a figure with relaxation time (T_2n_) from the LF-NMR measurements are available in the supplementary materials.

## Sample Availability

Samples of the compounds are not available from the authors.
