# Supplementary material for: The Effect of α-, β- and γ-Cyclodextrin on Wheat Dough and Bread Properties
Source: Molecules. 2021 Apr 13;26(8):2242. doi: 10.3390/molecules26082242 (PMC8070397; doi:10.3390/molecules26082242)

Supplementary materials

The Effect of α-, β- and γ-Cyclodextrin on Wheat Dough and Bread Properties

Anne-Sophie Schou Jødal [1,2], Tomasz Pawel Czaja [3,4], Frans W. J. van den Berg [3], Birthe Møller Jespersen [3] and Kim Lambertsen Larsen [1,*]

[1] Section of Chemistry, Department of Chemistry and Bioscience, Aalborg University, DK-9220 Aalborg, Denmark; asj@bio.aau.dk (A.-S.S.J.)
[2] Lantmännen Unibake Denmark, DK-8700 Horsens, Denmark
[3] Department of Food Science, Faculty of Science, University of Copenhagen, DK-1958 Frederiksberg, Copenhagen, Denmark; tomasz.czaja@food.ku.dk (T.P.C.); fb@food.ku.dk (F.W.J.v.d.B.); bm@food.ku.dk (B.M.J.)
[4] Department of Chemistry, University of Wrocław, 50-383 Wrocław, Poland
* Correspondence: kll@bio.aau.dk; Tel: +45-9940-8521

Pictures of cyclodextrin-supplemented breads (from Breadmaking I)

0 % cyclodextrin

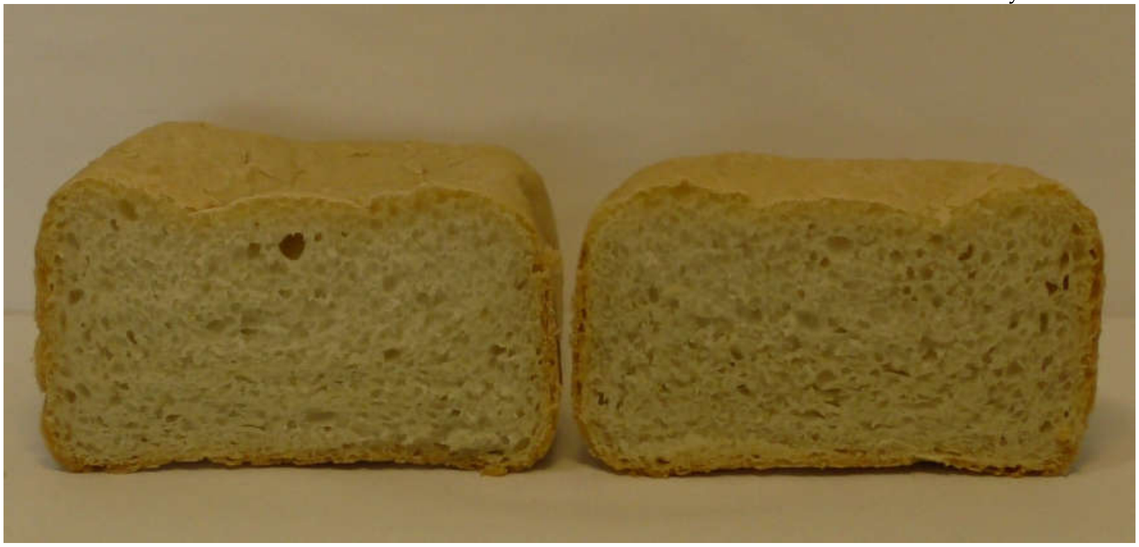

1 % α-cyclodextrin

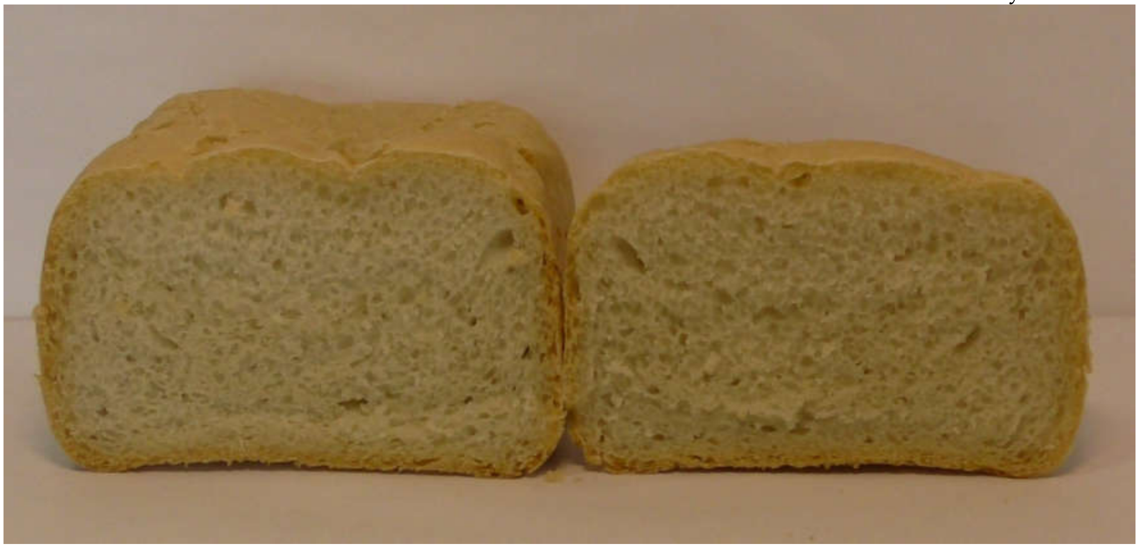

2 % α-cyclodextrin

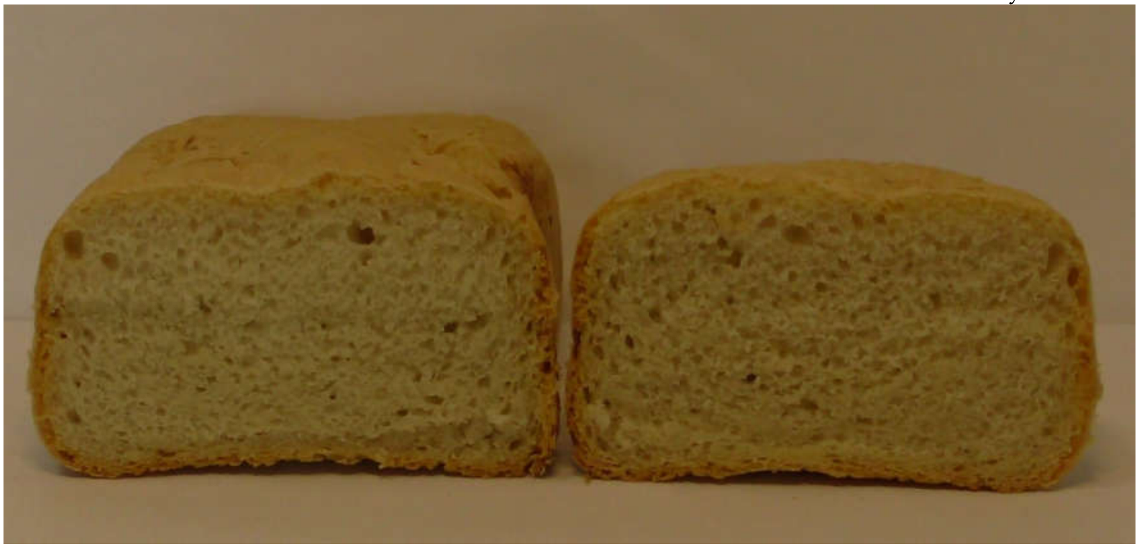

4 % α-cyclodextrin

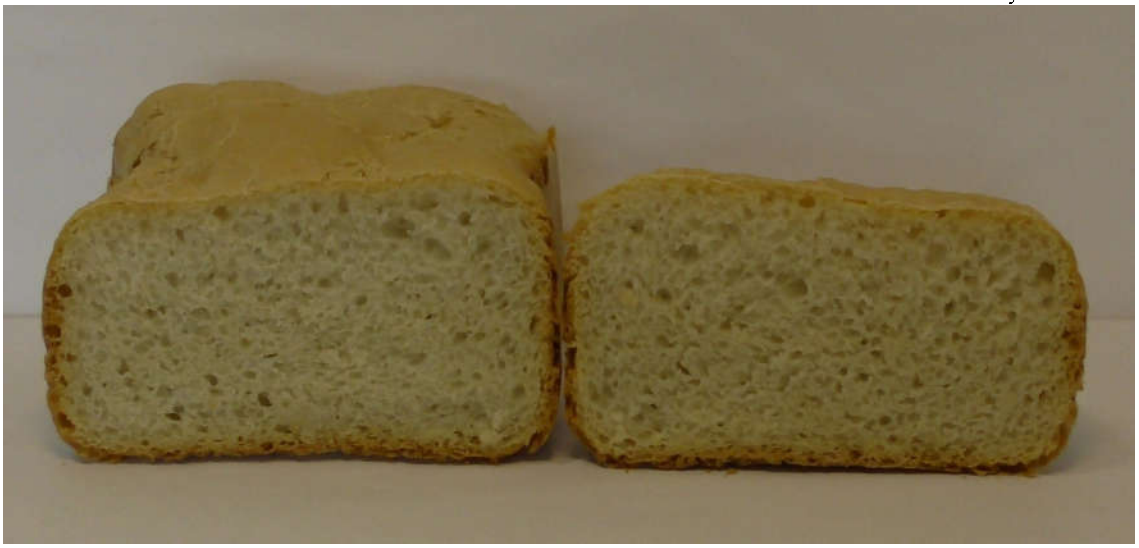

8 % α-cyclodextrin

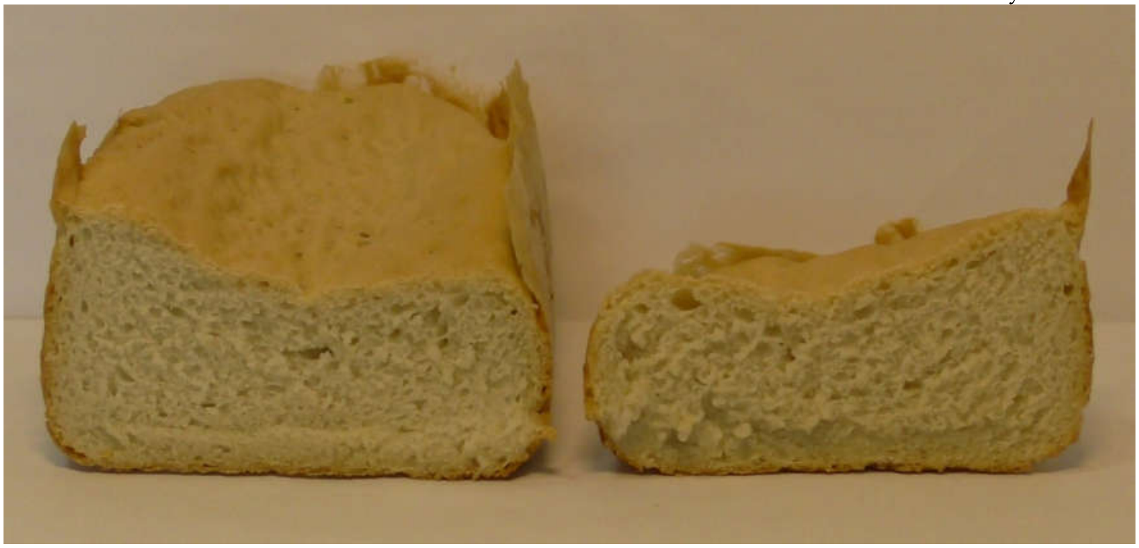

1 % β-cyclodextrin

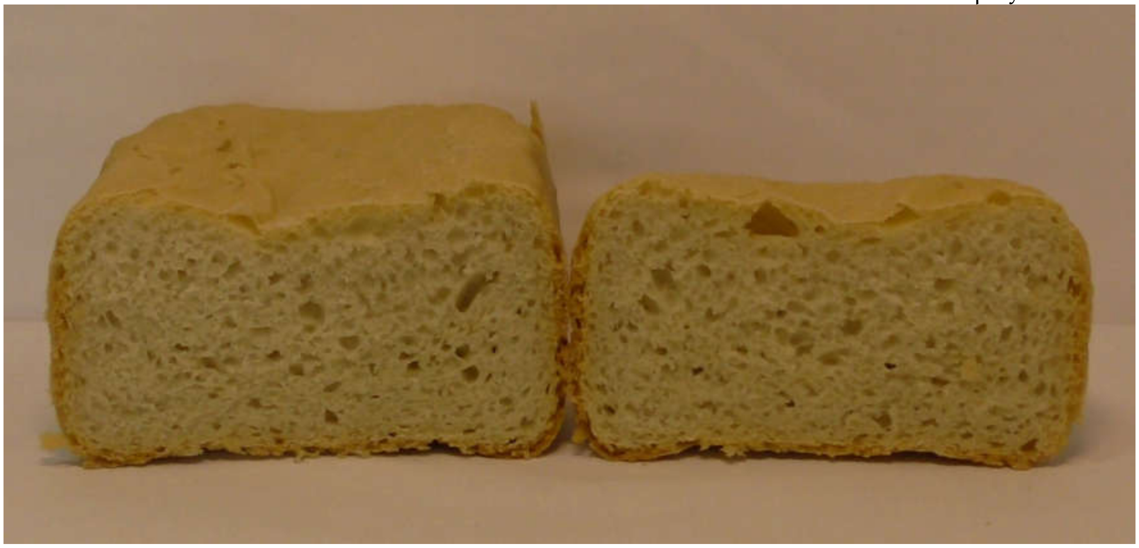

2 % β-cyclodextrin

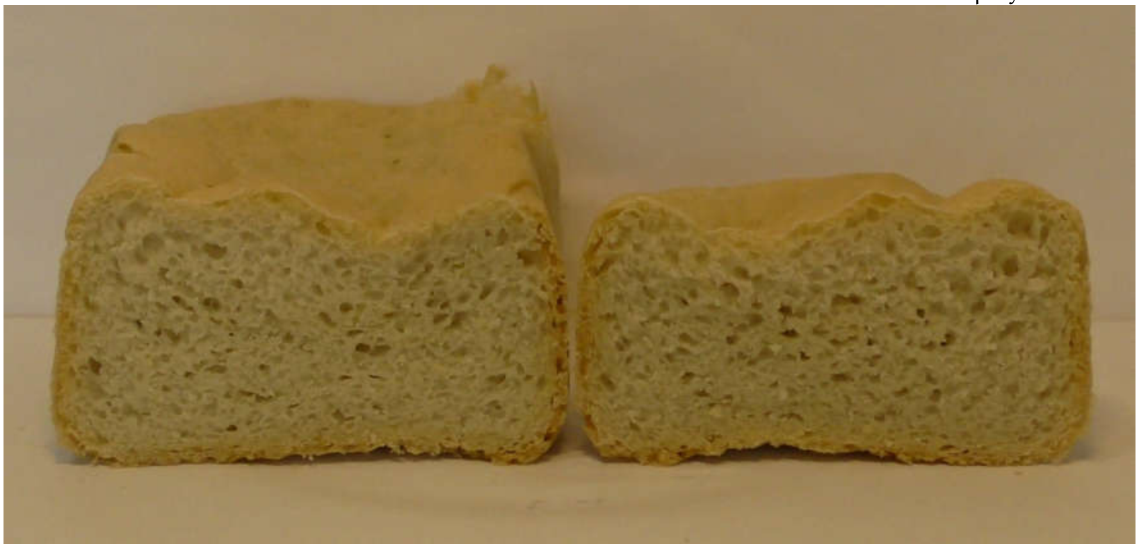

4 % β-cyclodextrin

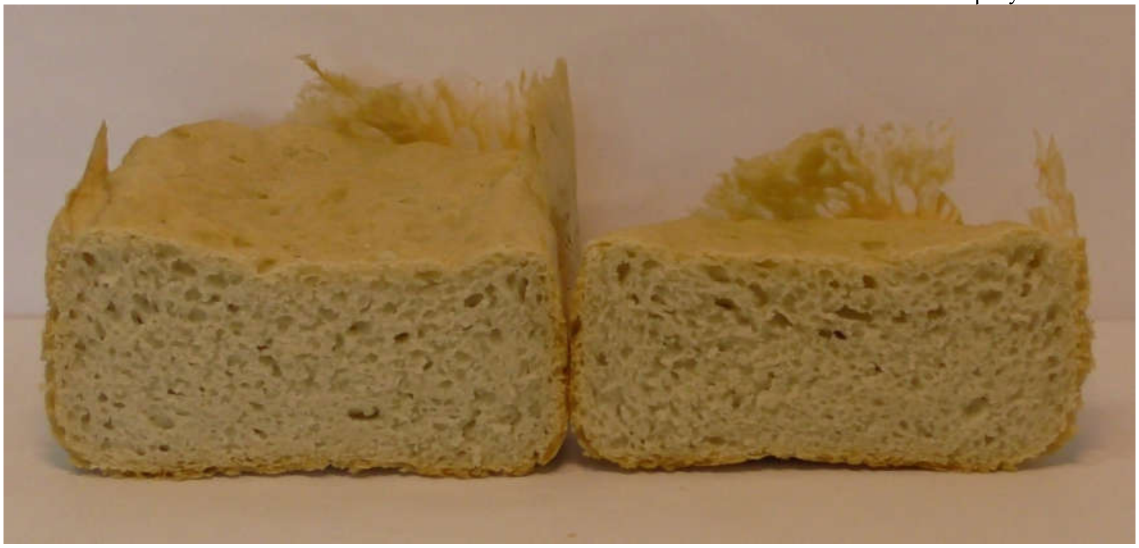

8 % β-cyclodextrin

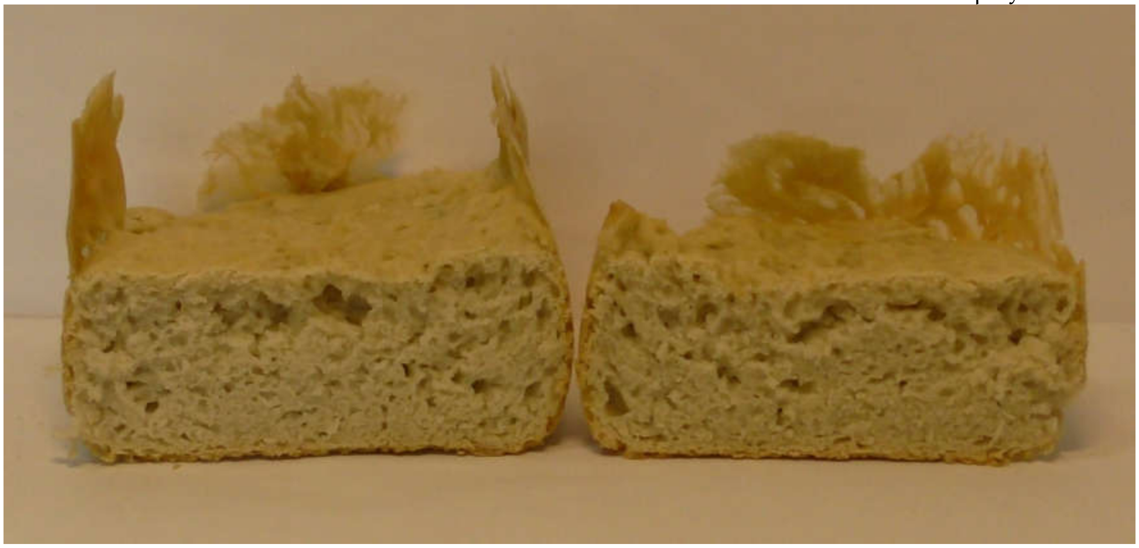

1 % γ-cyclodextrin

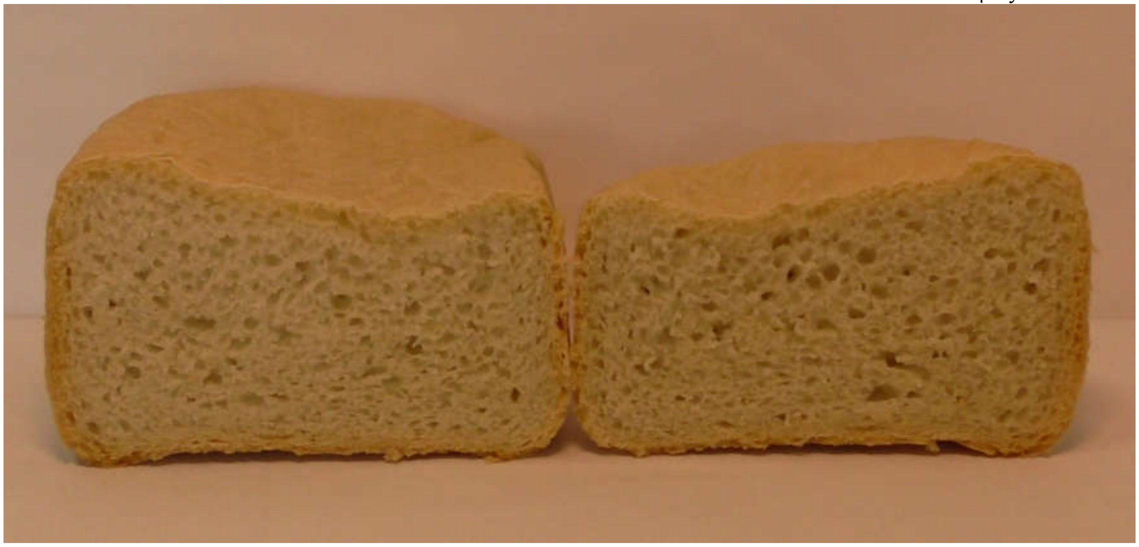

2 % γ-cyclodextrin

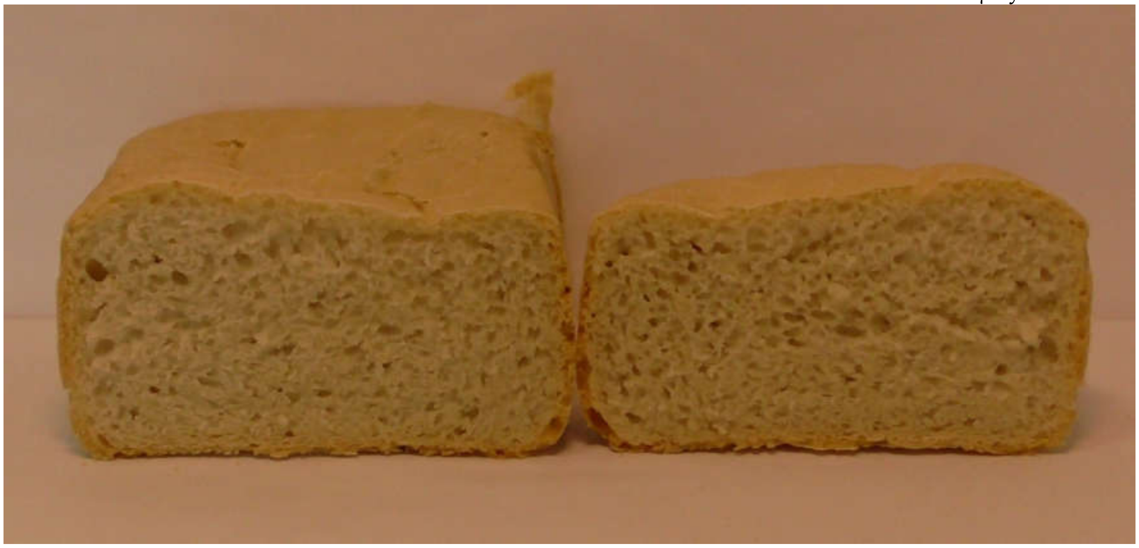

4 % γ-cyclodextrin

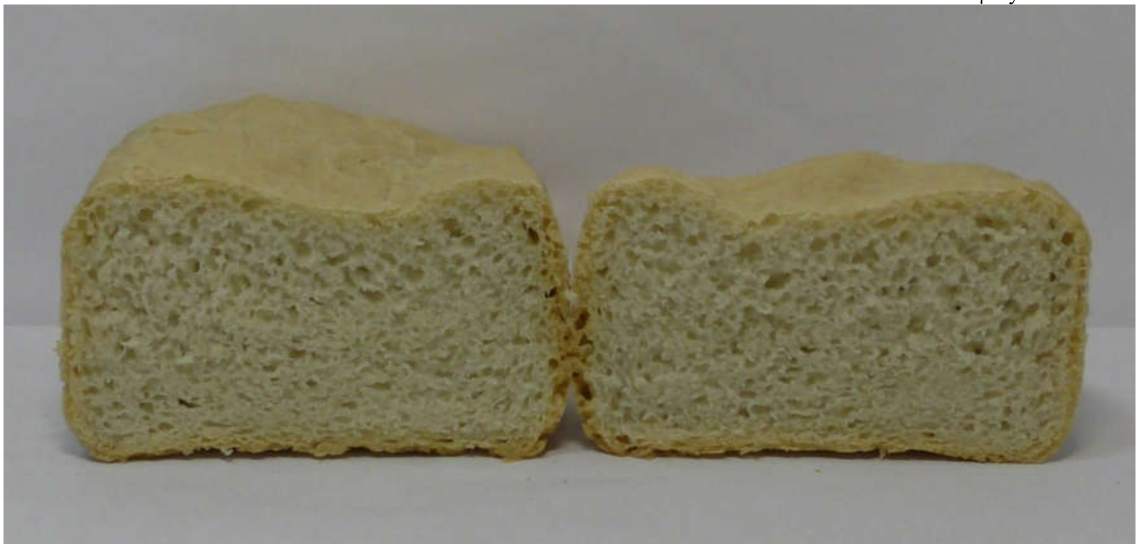

8 % γ-cyclodextrin

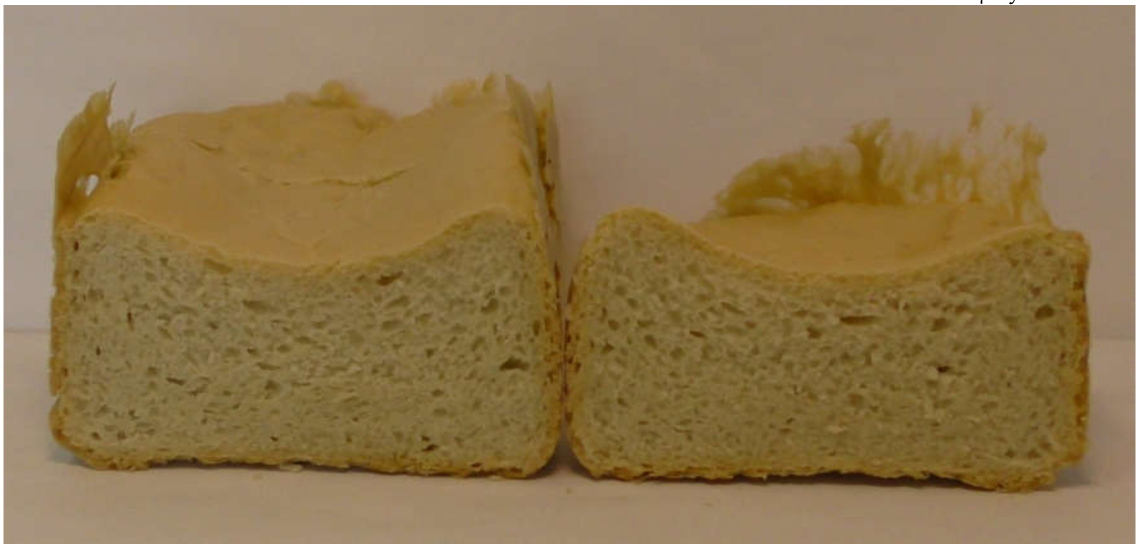

Relaxation time (T_{2n}) of the three different proton populations in the breads with different types and concentrations of α-CD (a), β-CD (b) and γ-CD (c) CDs for different storage time. The error bars indicate the standard deviation.

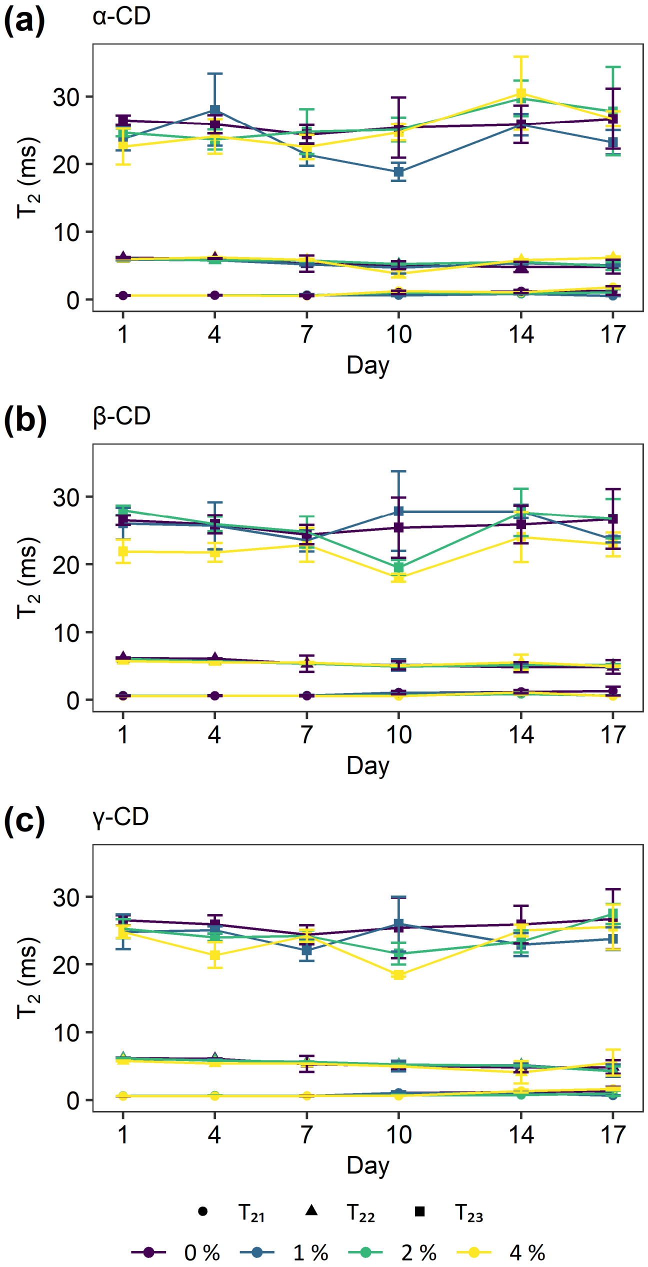

Supplement: Supplementary file 1 [file molecules-26-02242-s001.pdf]
